# The genetic landscape of intellectual disability and epilepsy in adults and the elderly: a systematic genetic work-up of 150 individuals

**DOI:** 10.1038/s41436-021-01153-6

**Published:** 2021-04-28

**Authors:** Pia Zacher, Thomas Mayer, Frank Brandhoff, Tobias Bartolomaeus, Diana Le Duc, Martin Finzel, Anja Heinze, Susanne Horn, Chiara Klöckner, Gudrun Körber, Julia Hentschel, Malgorzata Kalita, Ilona Krey, Marina Nastainczyk-Wulf, Konrad Platzer, Johannes Rebstock, Bernt Popp, Mathias Stiller, Anne-Christin Teichmann, Rami Abou Jamra, Johannes R. Lemke

**Affiliations:** 1 Epilepsy Center Kleinwachau, Radeberg, Germany; 2 Institute of Human Genetics, University of Leipzig Medical Center, Leipzig, Germany; 3 Praxis for Human Genetics, Tübingen, Germany; 4 Institute of Forensic Medicine, University Hospital Halle, Halle (Saale), Germany; 5 Center for Rare Disearses, University of Leipzig Medical Center, Leipzig, Germany

## Abstract

**Purpose:**

Genetic diagnostics of neurodevelopmental disorders with epilepsy (NDDE) are predominantly applied in children, thus limited information is available regarding adults or elderly.

**Methods:**

We investigated 150 adult/elderly individuals with NDDE by conventional karyotyping, *FMR1* testing, chromosomal microarray, panel sequencing, and for unresolved cases, also by exome sequencing (*n*_single _= 71, *n*_trios _= 24).

**Results:**

We identified (likely) pathogenic variants in 71 cases (47.3%) comprising fragile X syndrome (*n* = 1), disease-causing copy number (*n* = 23), and single-nucleotide variants (*n* = 49). Seven individuals displayed multiple independent genetic diagnoses. The diagnostic yield correlated with the severity of intellectual disability. Individuals with anecdotal evidence of exogenic early-life events (e.g., nuchal cord, complications at delivery) with alleged/unproven association to the disorder had a particularly high yield of 58.3%. Screening for disease-specific comorbidities was indicated in 45.1% and direct treatment consequences arose in 11.8% of diagnosed individuals.

**Conclusion:**

Panel/exome sequencing displayed the highest yield and should be considered as first-tier diagnostics in NDDE. This high yield and the numerous indications for additional screening or treatment modifications arising from genetic diagnoses indicate a current medical undersupply of genetically undiagnosed adult/elderly individuals with NDDE. Moreover, knowledge of the course of elderly individuals will ultimately help in counseling newly diagnosed individuals with NDDE.

## INTRODUCTION

Genetic testing of neurodevelopmental disorders with epilepsy (NDDE) is mainly applied in pediatric settings, thus little information is available on the course and spectrum of symptoms in adults or elderly.

Seizures and cognitive deficits are symptoms of altered neuronal functioning in the central nervous system (CNS), explaining well why epilepsy is much more prevalent in individuals with intellectual disability (ID). The prevalence of epilepsy in individuals with ID is estimated to be 22%, increasing with the degree of ID.^1^ In many cases, the seizure disorder proves to be pharmacoresistent, especially in the presence of, e.g., structural or metabolic abnormalities of the CNS.

Advocating on behalf of patients with NDDE, the International League against Epilepsy (ILAE) published guidelines recommending a thorough etiologic investigation to enable the best possible treatment as well as aiding research on new therapeutic approaches.^2,3^

The aim of our study is to demonstrate how comprehensive genetic testing directly influences patient care and to encourage clinicians to consider panel or exome sequencing not only in children, but also in adult and elderly individuals with NDDE.

## MATERIALS AND METHODS

### Study population and inclusion criteria

We screened 150 adult/elderly individuals with epilepsy and ID (in accordance to the International Classification of Diseases, 10th revision) of unknown origin. Individuals were recruited between October 2017 and July 2019 at the Epilepsy Center Kleinwachau, Germany, which provides specialized in- and outpatient care to patients with epilepsy and other seizure-like disorders. Evaluation was based on in-house referral by treating specialists of psychiatry, neurology/epileptology, or internal medicine. Also, family relatives, legal guardians, or health-care workers were encouraged to mention previously suspected clinical diagnoses.

All individuals with ID of unknown cause (IQ ≤ 70) and epilepsy were evaluated by an epileptologist with a background in clinical genetics. A detailed patient history was taken, physical examination was performed, and if available, medical records were re-evaluated. The possibility of genetic testing was offered to patients and/or legal guardians (Supplement 1—Methods A).

Study participants or their legal guardians gave their written informed consent. The study was approved by the ethics committee of the University of Leipzig, Germany (224/16-ek and 402/16-ek).

In total, we report on 150 adults with NDDE (72 females, mean age 46 years, ranging from 18 to 84 years). None were of known consanguineous origin. The vast majority of individuals were of German descent, whereas three probands originated from Central Asian countries (Azerbaijan, Kazakhstan, Afghanistan).

### Documented clinical data

We documented the degree of ID (according to ICD10), epilepsy type (including seizure types and age of onset), and electroencephalography (EEG), as well as previous cerebral imaging results, i.e., magnetic resonance imaging (MRI). Information regarding pregnancy, birth (including weight, length, head circumference), early development, relevant comorbidities, and family history was collected. Neurological, psychomotor, and psychiatric findings were documented as well as previous seemingly nongenetic diagnoses that were considered causative for the individual NDDE. In addition, each proband underwent a detailed physical examination at the time of recruitment as well as standardized photo documentation including facial photographs in case of consent.

### Diagnostic algorithm

Blood samples were taken from 150 individuals and in 42 cases also from both parents. Genetic testing comprised conventional karyotyping, *FMR1* testing, chromosomal microarray (CMA) testing, and comprehensive panel sequencing (TruSight One panel, Illumina) for every proband. Cases remaining inconclusive with these methods underwent additional single or trio-based exome sequencing (Supplement 1—Methods B). Detected single-nucleotide variants (SNVs) were evaluated according to the guidelines of the American College of Medical Genetics and Genomics-Association for Molecular Pathology (ACMG-AMP),^4^ validated by Sanger sequencing and if possible, segregation analysis was performed in parental blood samples. Finally, panel and exome sequencing data were utilized for coverage-based analysis of copy-number variants (CNV) in addition to previous CMA. Detected CNVs were evaluated according to ACMG guidelines and ClinGen.^5,6^

A clinical case was considered solved if the individual carried a pathogenic or likely pathogenic variant with association to its phenotypic spectrum.

## RESULTS

### Diagnostic yield depending on diagnostic method

For 71 of 150 individuals (47.3%, average age 41 years) a genetic NDDE diagnosis was identified (43 pathogenic, 32 likely pathogenic variants; including individuals with recessive or multiple diagnoses) and clinically confirmed by retrospective phenotyping.

Conventional karyotyping revealed three individuals with causative numerical or structural aberrations among the 150 probands (2.0%) as well as one secondary finding. Two individuals carried a small supernumerary marker chromosome of unknown origin and one individual had an unbalanced translocation (Supplement 2). All cytogenetically visible numerical or structural chromosomal aberrations were also detected by CMA and CNV analysis of next-generation sequencing (NGS) data. The unbalanced translocation was not detected at first sight during primary cytogenetic karyotyping, but it was visible after CMA analysis triggered re-evaluation. In a female individual, a Turner mosaic (22% of cells with karyotype of 45,X) was detected without any clinical symptoms of Turner syndrome. It was thus considered as a secondary finding. Further genetic testing was unable to reveal the etiology of the underlying NDDE in this female individual. In 147 of 150 individuals (98.0%) chromosomal analysis was unable to reveal the genetic background of the respective NDDE.

*FMR1* testing revealed one individual with a causative full mutation among 150 (0.7%) as well as one with the secondary finding of a premutation. One 61-year-old male exhibited >280 CGG repeats and a phenotype compatible with fragile X syndrome (MIM 300624) (Supplement 1 and 2) and no additional pathogenic findings were detected by NGS. One 19-year-old female was identified with a premutation of 58 CGG repeats, which was not considered causative for the etiology of the individual’s phenotype. Further panel diagnostics revealed a likely pathogenic variant in *ARID1B* leading to the diagnosis of Coffin–Siris syndrome type 1 (MIM 135900) in this young woman, which also was in agreement with her phenotype (Supplement 2). In 149 of 150 individuals (99.3%) *FMR1* testing was unable to reveal the genetic background of the respective NDDE.

CMA revealed 24 individuals with (likely) disease-causing CNVs (16%), including all 3 cases with abnormal conventional karyotyping but also including 2 cases with CNVs that only partially explain the respective NDDE phenotypes (deletion of 7p14.3 incl. *GARS*; deletion of 17p12 incl. *PMP22*).

These 24 individuals comprised 12 well-known recurrent microdeletions or microduplications, as well as 12 cases with unique CNVs (Supplement 2). In two individuals the respective (likely) pathogenic CNV co-occurred with other (likely) pathogenic variants that were primarily considered causative for the complex phenotypes of the respective individuals (Supplement 1-CSS5).

All CNVs detected by CMA were also detected via CNV analysis of NGS data. Through CMA, both cases of supernumerary marker chromosomes of unknown origin could be specified as 15q11-q13 duplication syndrome (MIM 608636). One of these two individuals displayed an unusually shaped marker chromosome with two additional copies of the region 15q11.1–15q12 (7.8 Mb) and two distal fluorescence in situ hybridization (FISH) signals of the LSI probe SNRPN separated by only one signal for centromere 15 (Supplement 1).

Four individuals were diagnosed with a 15q13.3 microdeletion syndrome (MIM 612001). All four exhibited combined generalized and focal epilepsy as well as mild cerebellar or cerebral atrophy without epileptogenic lesion on MRI. In 128 of 150 cases (85.3%) CMA was unable to unravel the genetic background of the respective NDDE.

NGS diagnostics revealed 50 individuals with NDDE-causing variants as well as four individuals with secondary findings. All NDDE-causing CNVs previously detected by CMA were also detected by CNV analysis of NGS data.

Panel sequencing was performed in all 150 individuals and revealed pathogenic or likely pathogenic NDDE-causing SNVs in 34 of 150 individuals (22.7%). Segregation analysis could be performed in 17 cases and revealed 13 individuals with heterozygous de novo variants, 3 individuals with postzygotic (mosaic) variants, and 6 individuals with inherited pathogenic or likely pathogenic SNVs including one case with a maternal mosaic of 35% variant allele frequency in maternal blood. Panel-based SNV analysis was unable to identify the genetic etiology of the respective NDDE in 116 of 150 individuals (77.3%).

After conventional karyotyping, *FMR1* testing, CMA, as well as panel sequencing, 57 of 150 individuals (38.0%) had a pathogenic or likely pathogenic finding that was considered causative for the respective phenotype. However, 93 of 150 remained unsolved or only partially solved and were thus processed by exome sequencing of singletons or (if available) parent–offspring trios.

Exome sequencing of singletons was performed in 71 individuals and revealed NDDE-causative variants in an additional 13 cases of the overall cohort of 150 individuals (8.7%, 12 individuals with SNVs and 1 individual with a CNVs) located in known disease genes outside the design of the TruSight One panel.

In one case, exome sequencing revealed only a single heterozygous pathogenic *CEP290* splice variant (c.6135 + 1G>A) that appeared compatible with the phenotype. Subsequent targeted Sanger sequencing additionally revealed the well-known pathogenic deep-intronic *CEP290* founder variant c.2991 + 1655G>A in compound heterozygous state, confirming the diagnosis of autosomal recessive *CEP290*-associated NDDE with visual impairment in this individual.

Exome sequencing of parent–offspring trios was performed in 24 cases and revealed NDDE-causative variants in additional 3 cases of the overall cohort of 150 individuals (2.0%). These cases comprise two individuals with de novo SNVs in novel disease genes (*KDM5A*,^7^ *TFE3*^8^). One additional individual carried a maternally inherited, likely pathogenic variant in *DEPDC5*, outside the design of the TruSight One panel. In addition, multiple de novo variants in genes of uncertain significance have been detected and will remain subject to further investigation.

Overall, NGS analysis identified (likely) pathogenic NDDE-causing SNVs in 49 individuals. In 18 of 49 individuals (36.7%) a de novo SNV was confirmed (including 3 individuals with mosaics). Nine of 49 individuals (18.4%) had inherited disease-causing variants (comprising 4 cases with autosomal recessive disorders, 1 individual with an SNV inherited via a parental mosaic, 2 individuals with X-linked disorders, and 2 individuals with variants associated with disorders known to be of reduced penetrance, each inherited from healthy parents).

CNV analysis of NGS data further increased the overall diagnostic yield. In one individual, panel sequencing revealed a *MSH6* deletion, which initially was considered a secondary finding. After exome sequencing (singleton), the deletion was identified to affect not only exons 5–10 of *MSH6* but also exons 18–23 of the neighboring gene *FBXO11*. Null variants in *FBXO11* are associated with a syndromic form of NDDE (MIM 618089). Likely due to the rather small size of 10.5 kb and sparse coverage of microarray probes at that locus, previous CMA had failed to detect this deletion (Supplement 1).

NDDE-causing SNVs in *ANKRD11*, *CUX1*, *DEPDC5*, *DYNC1H1*, *GNB1*, *KCNB1*, *MECP2*, *PAFAH1B1*, *STXBP1*, as well as invdup15 syndrome were each detected twice; *SCN1A* and *SLC2A1* in three individuals. The most common diagnosis was 15q13.3 microdeletion syndrome (4 of 150 individuals, 2.7%).

When disregarding one detected *FMR1* premutation as well as secondary findings,^9^ 4 of 150 individuals (2.7%) carried multiple causative variants leading to complex forms of NDDE. Three of these individuals were identified to have two and one individual to have three combined genetic disorders (Supplement 1-CSS5).

All detected variants were uploaded to ClinVar (Supplement 2).

### Geneotype/phenotype correlations of the study population

**Fig. 1:**
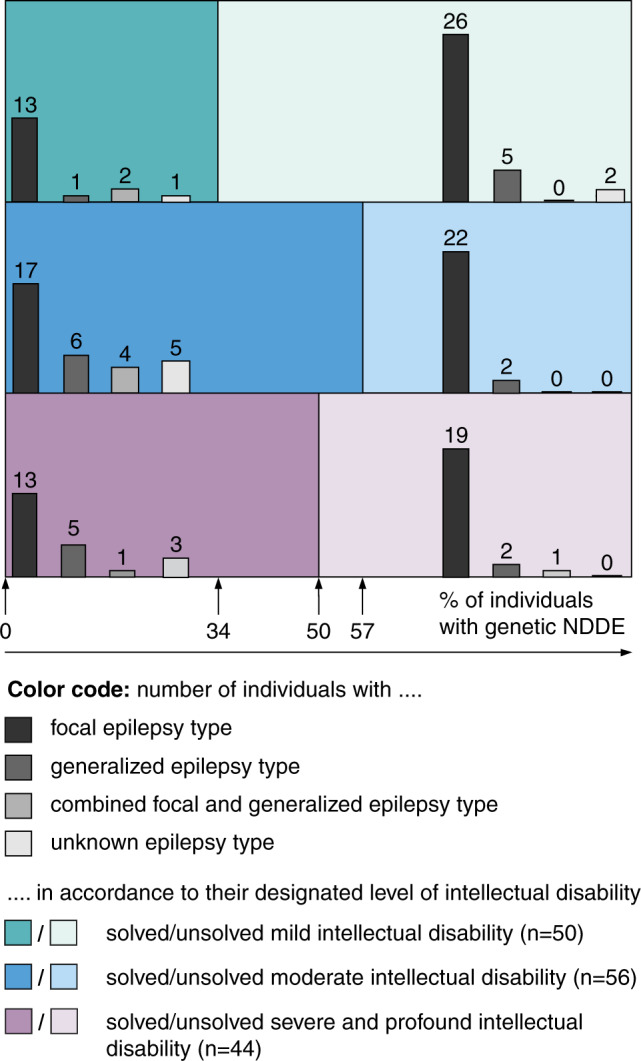
Study cohort of 150 individuals with neurodevelopmental disorders with epilepsy (NDDE), grouped according to their level of intellectual disability and respective epilepsy syndrome; (Ø 46 years, 18–84 years of age). Solved cases are depicted in solid color (left), whereas unsolved cases are in pastel (right). In total, 109 individuals exhibited focal epilepsy (*n*_mild ID_ = 39 of 50, *n*_moderate ID_ = 39 of 56, and *n*_severe/profound ID_ = 32 of 44). Generalized epilepsy (*n*_mild ID_ = 6 of 50; *n*_moderate ID_ = 8 of 56; *n*_severe/profound ID_ = 7 of 44) or combined focal and generalized epilepsy (*n*_mild ID_ = 2 of 50; *n*_moderate ID_ = 4 of 56; *n*_severe/profound ID_ = 2 of 44) were less frequent. In 11 individuals (*n*_mild ID_ = 3 of 50; *n*_moderate ID_ = 5 of 56; *n*_severe/profound ID_ = 3 of 44) too few data on the seizures disorder were available to categorize the epilepsy type according to the current International League against Epilepsy (ILAE) recommendations^23^.

Fifty of 150 individuals (33.3%) exhibited mild ID (ICD10 F70; 25 females), 56 individuals (37.3%) had moderate (ICD10 F71; 30 females), and 44 (29.3%) had severe or profound ID (ICD10 F72/F73; 17 females). The diagnostic yield varied and was increased in individuals with more severe ID as depicted in Fig. 1 (*n*_(mild ID)_ = 17 of 50 cases [34.0%], *n*_(moderate ID)_ = 32 of 56 cases [57.1%], and *n*_(severe/profound ID)_ = 22 of 44 cases [50.0%]; chi-square value *χ*^2^ of 6.3 [critical limit of 5.99], Supplement 4).

Cerebral imaging data were available in 143 of 150 individuals (95.3%) comprising 136 individuals with cerebral MRI, 6 individuals with cerebral computed tomography (CT), and 1 individual with antiquated pneumoencephalography. Cerebral imaging was normal in 33 of 143 individuals. In 18 of these 33 individuals (54.5%) a genetic diagnosis was identified.

Cerebral imaging detected unspecific or subcortical anomalies in 63 of 143 individuals (44.1%) including cerebral or cerebellar atrophy, periventricular leukomalacia, and dysgenesis of subcortical structures. In 32 of these 63 cases (50.8%) a genetic diagnosis was identified.

Cerebral imaging revealed cortical pathologies in 47 of 143 individuals (32.9%) with 17 genetic diagnoses among these 47 (36.2%). These 47 cases with cortical pathologies comprised 24 individuals with diffuse or focal gliotic lesions involving cortical structures (including 7 cases with genetic diagnoses), 20 individuals with cerebral malformations (disturbed neuronal migration, neuronal proliferation or cortical organization)^10^ (including 8 cases with genetic diagnoses), as well as 3 individuals with either an unspecified lesion of the occipital cortex, pilocytic astrocytoma, or meningioma (including 2 cases with genetic diagnoses).

One female individual presented with evidence of iron accumulation in the basal ganglia in the likely context of a β-propeller protein-associated neurodegeneration (MIM 300894). Her MRI represents the rare case of being suggestive for a particular genetic diagnosis.

In 76 of 150 probands (50.7%, average 42 years of age) anamnestic data had previously attributed the NDDE to nongenetic external circumstances/exogenic factors that were retrospectively not verifiable. These included 52 cases of alleged pregnancy- or birth-related events (68.4%, e.g., suspected teratogenic medication, maternal infections during pregnancy, nuchal cord, preterm delivery) as well as 15 cases with alleged postnatal events (19.8%, e.g., alleged CNS infection, CNS trauma, suspected cerebral hemorrhage). For 9 of 76 individuals (11.8%) a combination of such pre- and postnatal events had in the past been considered causative for the respective NDDE. However, the provided clinical data of all these individuals appeared insufficient to rely on the potential causality of these exogenic factors as, e.g., cerebral imaging did not support the proposed pathology and/or lumbar puncture at time of alleged CNS infection did not support the diagnosis of infection and/or there was clear evidence for a syndromal phenotype due to, e.g., dysmorphism and/or congenital malformation.

Overall, in 41 of these 76 individuals (53.9%), a genetic diagnosis was made independently of previously suspected exogenic factors. This proportion was even higher among the 48 of 76 individuals that had normal cerebral imaging, where 28 of these 48 individuals (58.3%) carried a (likely) pathogenic variant (case-specific Supplement 1) supporting a genetic rather than an exogenic cause of the NDDE.

Febrile seizures are the most common and usually benign pediatric seizure syndrome, clearly separated from epilepsy, affecting approximately 2–5% of children before the age of 5 years.^11^ Febrile seizures were reported in 21 of 150 individuals (14.0%). For 12 of those 21 individuals (57.1%) a genetic NDDE was identified (*ARX*, *DYNC1H1*, *EEF1A2*, *KDM5A*, *PCDH19*, *SCN1A*, *PTEN*, microdeletion 1q44, and microduplication 16p13.11 plus microdeletion 16p12.2).

### Secondary findings

In total, 117 of 150 individuals (78.0%) chose to be informed of secondary findings^9^ of whom four (3.4%) had a relevant secondary diagnosis. These four individuals carried one of the four following actionable (likely) pathogenic variants: *BRCA2* (c.5303_5304del, p.[Leu1768Argfs*5]), *MYPBC3* (c.26–2A>G, p.?), *DSG2* (c.1232_1354del, p.[Glu446*]), as well as a deletion of *MSH6* (c.3173–239_*6835del, p.?) as part of a continuous gene deletion syndrome. All individuals were currently asymptomatic for disease-associated symptoms related to these secondary findings.

### Consequences of successful phenotyping

In 32 of 71 individuals who received a NDDE diagnosis (45.1%), a more detailed evaluation regarding disease-specific comorbidities was recommended. These involved screening for disease-associated malformations and/or cardiac arrhythmias and/or potential tumor predispositions that had previously been associated with the respective disorder—even if the individual evidence remained low.

We evaluated disease-associated treatment approaches and recommendations according to the levels of evidence suggested by the Oxford Centre of Evidence-based Medicine (CEBM) in 2009.^12^ We modified CEBM levels to include logical clinical deduction and marked such accordingly (Supplement 3). In total, 76 individuals were diagnosed with genetic disorders (including 71 individuals with clear genetic cause of their NDDE as well as 5 individuals with only partial genetic clarification of the phenotype or with solely secondary findings). In 9 of 76 individuals (11.8%), the highest CEBM levels (I and II) were applicable and enabled precision medicine approaches. Respective diagnoses comprised individuals with *SCN1A*-related disorder, *SLC2A1*-related disorder, as well as individuals with secondary findings. For another 23 of 76 individuals (30.3%), moderate CEBM level III was applicable suggesting potential or future precision medicine approaches that still require additional supportive evidence before implementation in general treatment recommendations. Respective diagnoses were related to the following genes or disorders: *ANKRD11*, *CEP290*, *CHD2*, *DEPDC5*, *DNMT3A*, *FMR1*, *GNAS*, *GRIN2A*, *MECP2*, *MITF*, *MYT1L*, *NPR2*, *NPRL3*, *PCDH19*, *PTEN*, *SCN2A*, *SLC6A8*, *TSEN54*, *WDR45*, *MECP2* duplication syndrome, 22q11.2 deletion syndrome, 17p12 deletion syndrome (incl. *PMP22*).

## DISCUSSION

We identified the underlying genetic etiology of 71 of 150 individuals with NDDE (47.3%). This diagnostic yield comprises 23 individuals with CNV (15.3%), 49 with SNV (32.7%), and 1 with *FMR1*-CGG repeat expansion (0.7%) and includes 2 individuals with combined SNV and CNV diagnosis (1.3%).

Compared with previously published studies, our diagnostic yield of 47.3% is considerably higher than the approximately 23% of panel-based approaches focusing on primarily SNVs and disregarding CNVs.^13,14^ The yield of SNV rises when the sequencing target is increased from panel to whole exome, achieving yields between 25% and 38%.^15–17^ A recent study investigated both SNV and CNV within exome sequencing data and revealed a diagnostic yield of 31% (28/96 SNV and 2/96 CNV).^18^ Whereas the SNV detection rate is in line with the above studies, including ours, the CNV yield of 2.1% is for unknown reasons less than would have been expected, as array-based techniques have previously been published to achieve yields of 12.7–16.1%.^19,20^ Our data confirm these previously published yields and show that CNV analysis simply adds to the yield of SNV, allowing for the detection of genetic diagnoses of nearly half of 150 adult patients with NDDE in our cohort.

Considering solely the 71 individuals with conclusively clarified NDDE diagnoses, the yield of conventional genetic testing methods alone appeared to be rather low: conventional karyotyping (3/150; 2.0%), *FMR1* testing (1/150; 0.7%), CMA (22/150, 14.7%). By contrast, NGS-based analysis (of both SNVs and CNVs) had by far the highest diagnostic yield (69/150; 46.0%) and depicted almost all cases where a disease-causing variant had been revealed with any method (69/71; 97.2%). Only one case of *FMR1* trinucleotide expansion was not detected and an additional case with *CEP290*-associated NDDE was only partially clarified by NGS, due to nondetection of a deep-intronic second variant outside the enriched target sequence. Reassuringly, 100% of NDDE-causing cytogenetic aberrations had also been identified by CMA and 100% of CNVs from CMA had also been identified by NGS-based CNV analysis.

We therefore argue that NGS-based sequencing techniques, especially exome sequencing, should be considered as first-tier diagnostics in NDDE as it has the highest diagnostic yield. It is also capable of detecting most (if not all) alterations that are usually revealed by conventional genetic testing methods, such as cytogenetic karyotyping and CMA. To further slightly increase the diagnostic yield in a second step, sequential *FMR1* analysis can be considered. This hypothesis is further supported by Borch et al.,^21^ who suggest transitioning *FMR1* testing to a second-tier test if children with neurodevelopmental delay lack suggestive clinical features or family history. However, conventional genetic testing, such as cytogenetic karyotyping, CMA, as well as targeted Sanger sequencing should no longer be considered for primary screening of genetic causes in NDDE. However, these methods may remain important for confirmation and validation of NGS findings, e.g., validation of a supernumerary marker chromosome as the cause of a partial duplication detected in NGS-based CNV analysis.

The genetic background of an individual’s NDDE can be complex, as 4 of 150 probands (2.7%) carried multiple (likely) pathogenic variants, contributing to mixed or combined NDDE phenotypes. This further illustrates the preponderance of exome sequencing as this genetic complexity would likely not have been depicted by gene panel analysis with narrowed target.

The diagnostic yield correlated with the severity of ID as it was higher in individuals with moderate (57.1%) and severe to profound (50.0%) ID, compared with mildly (34.0%) affected individuals, which is in line with previous studies.^22^

Anecdotal evidence of alleged/unproven exogenic factors (e.g., teratogenic medication, maternal infections during pregnancy, nuchal cord, complications at delivery, CNS trauma) paradoxically appear to be a positive predictor for a genetic etiology of the respective NDDE in individuals with no or unspecific cerebral imaging changes, of whom 58.3% received a genetic diagnosis.

Additionally, febrile seizures had a threefold higher prevalence in our cohort of individuals with NDDE compared with the 2–5% in the general population.^11^ As our cohort size is likely too small to ultimately clarify this potential enrichment of febrile seizures among individuals with NDDE, this observation may need to remain the subject of further investigation.

The 3.4% yield of actionable secondary findings is in line with previous studies.^18^ In a population of individuals with ID, this aspect may be of particular importance. The more severe the cognitive and physical deficits of an individual, the less likely he or she may be able to realize as well as to articulate relevant disease-related symptoms (e.g., arrhythmic heartbeat, abnormal lymph nodes). Thus, the clinical self-detection of actionable secondary findings is likely decreased and may become considerably detrimental to individuals with ID. Additionally, the high number of individuals (32 of 71; 45.1%) where the genetic diagnosis indicated a more detailed evaluation regarding disease-specific comorbidities, as well as the 9 of 71 (11.8%) individuals with direct treatment consequences, illustrate a higher risk for medical undersupply of genetically undiagnosed individuals with NDDE.

Studying adult and elderly individuals with NDDE represents an important source of knowledge on particular types of NDDE. In no more than 150 cases, we describe a 31-year-old female with pontocerebellar hypoplasia type 2A (MIM 277470) as well as an 80-year-old male with GLUT1 deficiency (MIM 606777). To the best of our knowledge, both cases are the oldest known individuals with their respective type of NDDE and the individual with GLUT1 deficiency is likely also the oldest patient ever having been switched to a ketogenic diet. Since comprehensive data regarding phenotypes of adults and elderly are sparse, we provide detailed phenotypic descriptions for all individuals with clarified genetic NDDE diagnosis (Supplement 1 and 2).

We argue that genetic testing of individuals with NDDE frequently has direct implication on therapy—even in adult and elderly patients. Consequently, the fraction of individuals who might directly benefit will continue to grow, in the likely case that more evidence on approaches with lower CEBM levels can be collected in the years to come.

We therefore encourage clinicians to consider genetic testing in particular in adult and elderly individuals with NDDE. First and foremost, individuals with ID deserve a higher degree of medical attention independent of their age. Especially in assisted living facilities for people with ID, knowledge has been acquired about individuals that sometimes covers the longitudinal course of several decades. This knowledge may contain fundamental information on the course as well as on therapeutic responses of numerous individual types of rare NDDE. However, this knowledge is largely worthless if it cannot be associated with the respective etiologic diagnosis and will ultimately be lost upon the individual’s death, if he or she remained undiagnosed. Learning from these courses of elderly individuals with NDDE may help validate past treatment successes as well as avoid treatment errors. Moreover, it may help newly diagnosed infants and their families to be prepared for what to expect not only in the upcoming few years but also decades.

## Supplementary information

Supplement 1 - methods and case specific notes - clean

Supplement 2 - clinical case data

Supplement 3 - genotype phenotype correlation and therapy

Supplement 4 - chi-square calculations

## Data Availability

All data will be made available upon request.
