# Supplementary material for: The genetic landscape of intellectual disability and epilepsy in adults and the elderly: a systematic genetic work-up of 150 individuals
Source: Genet Med. 2021 Apr 28;23(8):1492–7. doi: 10.1038/s41436-021-01153-6 (PMC8354852; doi:10.1038/s41436-021-01153-6)
Supplement: Supplementary file 1 — Supplement 1 - methods and case specific notes - clean [file 41436_2021_1153_MOESM1_ESM.pdf]

Supplemental information 1

Table of contents

1. **Diagnostic methods** **page 2**
2. **Patient data and biases** **page 6**
3. **Case-specific supplement (CSS)** **page 8**
4. **Genetic diagnosis and postnatal exogenic factors** **page 12**
5. **Cases with multiple diagnoses** **page 13**
6. **References** **page 16**

1. Diagnostic methods

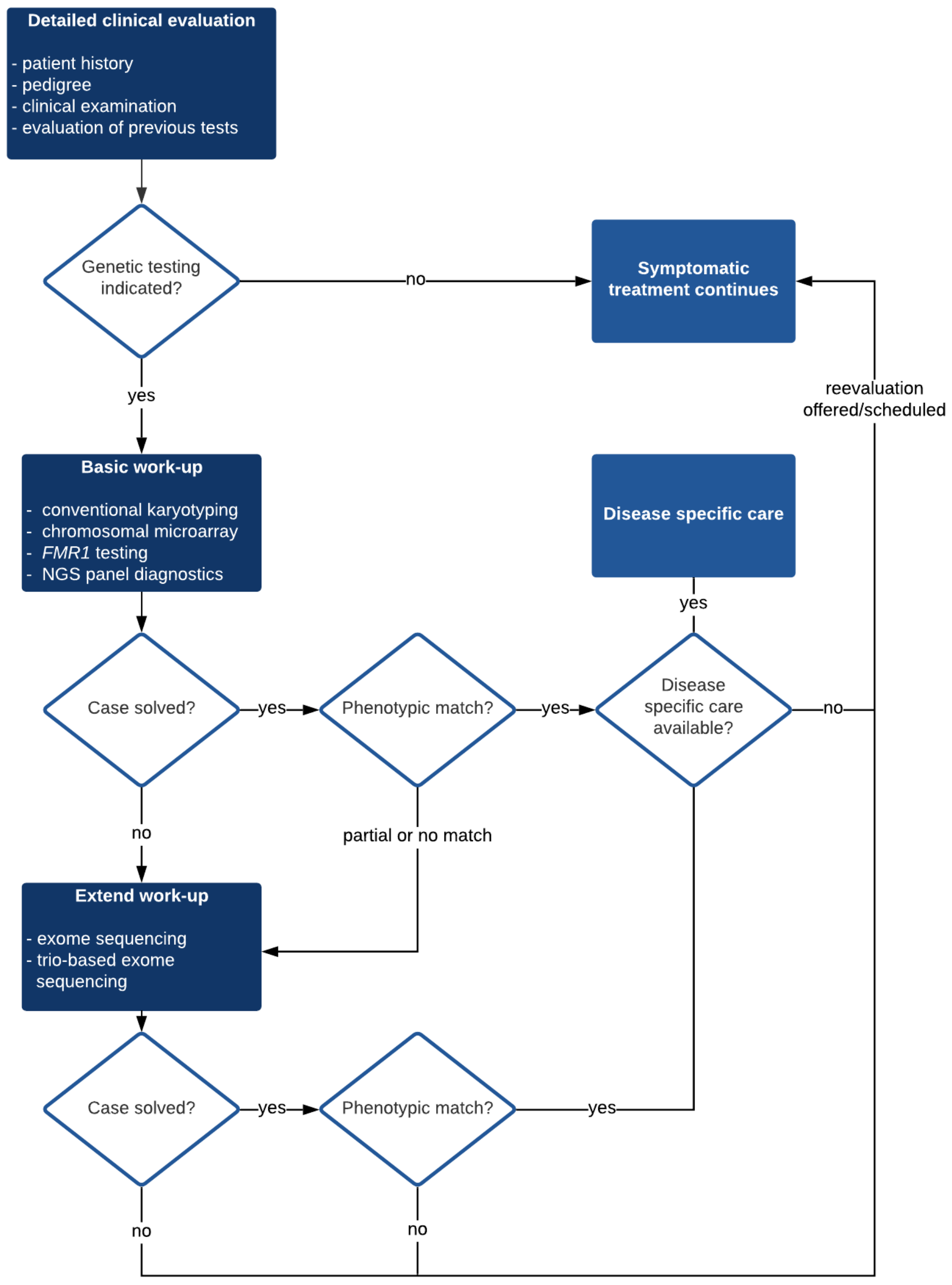

Figure 1: Diagnostic workflow: Individuals were recruited chronologically, according to their date of recruitment. Data analysis was performed after the first 150 recruited patients were fully evaluated.

Conventional cytogenetic karyotyping followed standard protocols, where lymphocyte cultures were incubated in ready-to-use Lympho Grow II medium, Chromosome Medium P (containing fetal calf serum plus phytohemoagglutinin) for 72h at 37°C. Colcemid was added 2h before culture termination. Chromosome preparation was aiming for a resolution of 550 bands per haploid set[2]. The chromosomes were banded by trypsin followed by Giemsa (GTG) and evaluated using IKAROS (MetaSystems, Altlussheim, Germany). In a few cases additionally fluorescence in-situ hybridization (FISH) was performed according to standard protocols for further evaluation of an abnormal cytogenetic result.

Microarray testing was performed by SNP array analysis using the Infinium CytoSNP-850K v 1.1. and 1.2 and a scan on the NextSeq 550 (all Illumina, Inc., San Diego, CA, USA). The effective resolution of the CytoSNP-850K is 18 kb (app. 1 SNP every 1.8 kb, ten SNPs are required for calling CNV gain/loss). Every gain or loss detected by BlueFuse Multi Software version 4.4 (Illumina, Inc., San Diego, CA, USA) was filtered against Decipher[3], ISCA[4] and the Database of Genomic Variants (DGV)[5].

FMR1 testing was performed for all probands, regardless of their gender, using AmplideX PCR/CE *FMR1* Kit. In cases of apparent homozygosity of CGG alleles, methylation analysis using AmplideX mPCR was applied (all Asuragen, Inc., Austin, TX, USA).

Next-Generation Sequencing (NGS) comprised panel, single exome as well as trio exome sequencing.

NGS targeted panel: DNA from peripheral blood lymphocytes (PBL) was extracted by standard procedures. Every individual included in this study initially received clinical panel sequencing (from now on called "NGS panel"). Enrichment and library preparation for the NGS panel were performed with the TruSight One v1 panel (May 2014 edition, 4811 genes, Illumina, Inc., San Diego, USA) according to the manufacturers' instructions. Resulting libraries were sequenced with 150 bp paired end reads on a NextSeq550 system (Illumina, Inc., San Diego, USA). For all included individuals, the genomic regions targeted by the NGS panel had an average coverage of > 100 reads and > 97% were covered by ≥ 20 reads.

NGS exome sequencing: For individuals with no diagnostic variant identified in the NGS panel, we next performed exome sequencing (ES). Enrichment and library preparation for ES was performed on PBL DNA using either the TWIST Human Core Exome Kit (TWIST Bioscience, San Francisco, CA, USA) or BGI Exome capture 59M kit (BGI, Shenzhen, China). TWIST libraries were sequenced with 150bp paired end reads on a NovaSeq 6000 system (Illumina, Inc., San Diego, CA, USA) while the BGI libraries were sequenced with 100bp paired end reads on a BGISEQ-500 system (BGI, Shenzhen, China). For all included individuals, the genomic regions targeted by the respective enrichment design had an average coverage of > 100 reads and > 97% were covered by ≥ 10 reads.

NGS bioinformatic data processing: The resulting raw sequencing data of NGS panel and ES in bcl format was processed using the cloud based "varfeed" pipeline (Limbus Medical Technologies GmbH, Rostock, Germany).

In a tertiary analysis we used the browser-based "Varvis" genomics software (Limbus Medical Technologies GmbH, Rostock, Germany) to evaluate SNVs/indels and CNVs on a case-by-case basis. Symptoms of each individual were annotated by the clinician using the medical history records and the database of Human Phenotype Ontology (HPO) terms provided in Varvis.

SNV/indel variants were first filtered according to in-house quality standards (read coverage ≥ 10 quality and an allele frequency ≥ 0.2). All possible modes of inheritance (sporadic *de novo*, dominant, recessive, X-linked) were analyzed using sensible minor allele frequency cutoffs (recessive and X-linked ≤ 1%, dominant ≤ 0.01%) in public population databases (gnomAD [6]). Further filtering steps included a query for variants in known disease genes with annotated HPO terms matching the terms annotated in the proband (≥ 2 terms) and a query for variants already described as pathogenic in the public ClinVar database[7].

Called variants were evaluated for clinical plausibility, inspected for quality using the IGV browser[8] as well as protein-influencing potential through computational ("*in silico*") prediction tools (e.g.

MutationTaster [9], Polyphen2 [10], GERP++ [11], PhyloP [12], SpliceSiteFinder-like [13], MaxEntScan [14], NNSPLICE [15], Human Splicing Finder [16] using typical cut-offs as recommended in the original publications.

Segmented copy-number (CN) calls from CNVkit [17] were filtered in the varvis tool by 1) their log2 ratios to identify CN-losses (deletions < -0.6 log2 ratio) and CN-gains (duplications > 0.7 log2 ratio) and 2) the "Refspread" of the segments in the pooled reference from the same run samples (threshold < 0.2). The annotated (allexes pipeline) CN segments were further filtered for overlap with variants from our in-house and the public DGV [5] and DECIPHER CN-databases [3]. Such identified CN-calls were inspected for quality using both the visualization of CNVkit segments and bins in varvis as well as the alignment profiles in IGV browser [8].

The quality of the sequencing was high and homogeneous, which is essential for the CNV analyses. For cases that were performed using TWIST exome, the average coverage was 129 with a standard deviation of 20. For the cases that were performed using BGI exomes, the average coverage was 135.5 and the standard deviation was a little higher at 26.5.

NGS data evaluation: Each set of NGS data was analyzed by two evaluators at minimum. The diagnostic evaluations during the first standardized basic work-up (chromosomal analysis, *FMR1* testing, chromosomal microarray and targeted NGS panel), and in a trio-based exome setting were performed by genetic scientists and clinical geneticists. The recruiting epileptologist and a clinical geneticist performed the evaluations of exome sequencing data sets of singletons, when no or just one parental sample was available.

2. Patient data and biases

Imaging data: The evaluation of cerebral imaging for potential epileptogenic cortical lesions is a skillset of radiologists/neuroradiologist, routinely requested by an epileptologist.

In the setting of the epilepsy center Kleinwachau, Germany, the evaluation of imaging data is often a combined effort of neuroradiologist and treating epileptologist, knowing (inter-)ictal EEG findings and seizure semiology. For the purpose of this study the written imaging reports from patient records were examined. The results may vary depending on each given skillset.

Anamnestic data was taken from available patient records and interviews with family, legal guardians, caretakers or probands themselves. If medical records regarding other plausible etiologies were available, they were reported as a fact in the clinical supplement. Evidence suggests that at least two out of 150 individuals exhibited a phenotype due to a combination of a confirmed genetic diagnosis and postnatal exogenic factors (supplement 1-CSS#4).

Biases: Regarding the cohort there is a strong selection bias, recruiting though just one tertiary center primarily caring for patients with refractory epilepsy. Additionally, only one epileptologist, who has worked in the field of clinical genetics and counselling before, was evaluating all probands and patient records at the time of inclusion. Epilepsy centers frequently care for patients with genetic syndromes. "Easy" to spot diagnosis (e.g. Trisomy 21, Tuberous Sclerosis etc.) are not part, and well defined clinical syndromes (e.g. Fragile X and Dravet syndrome) may possibly be depleted in the described cohort, as affected individuals may already have received a diagnosis by targeted genetic testing prior to this study.

Also, having the recruiting epileptologist perform the primary evaluation of singleton sequencing data under supervision of a clinical geneticists, may have aided the diagnostic yield. This kind of set-up will not be plausible for most institutions.

We noticed, that a smaller percentage of trio-based exomes were solved than exome singletons. This might be due to the recruiting clinician's bias in evaluating patient only exome data, and aggressively researching missense variants with phenotypic overlap (e.g. the reported *CEP290* case of individual #48). Another plausible human error detected, was working with *de novo* filters in a trio-based setting, potentially overlooking a parental mosaic state, as well as variable penetrance or phenotypes. Thirdly individuals undergoing trio-based sequencing were generally younger, belonging to an adult generation (average age of 33 years, 7 years less than those undergoing exome sequencing only). This age group possibly has had access to genetic testing before. It is therefore plausible, that individuals with easily detectable diagnoses were solved already in a pediatric setting, while the more challenging cases remained to be recruited for this study.

3. Case-specific supplement (CSS)

1. Fragile X syndrome (MIM#300624):

Due to a presentation including spastic tetraparesis, internal hydrocephalus with widened sulci, possibly a malformed Corpus callosum and small hippocampi bilaterally, exome sequencing was performed for individual #32 as well. No other potentially relevant change was detected. Anamnestic data suggested a preterm delivery.

2. 15q11-q13 duplication syndrome (MIM#608636)

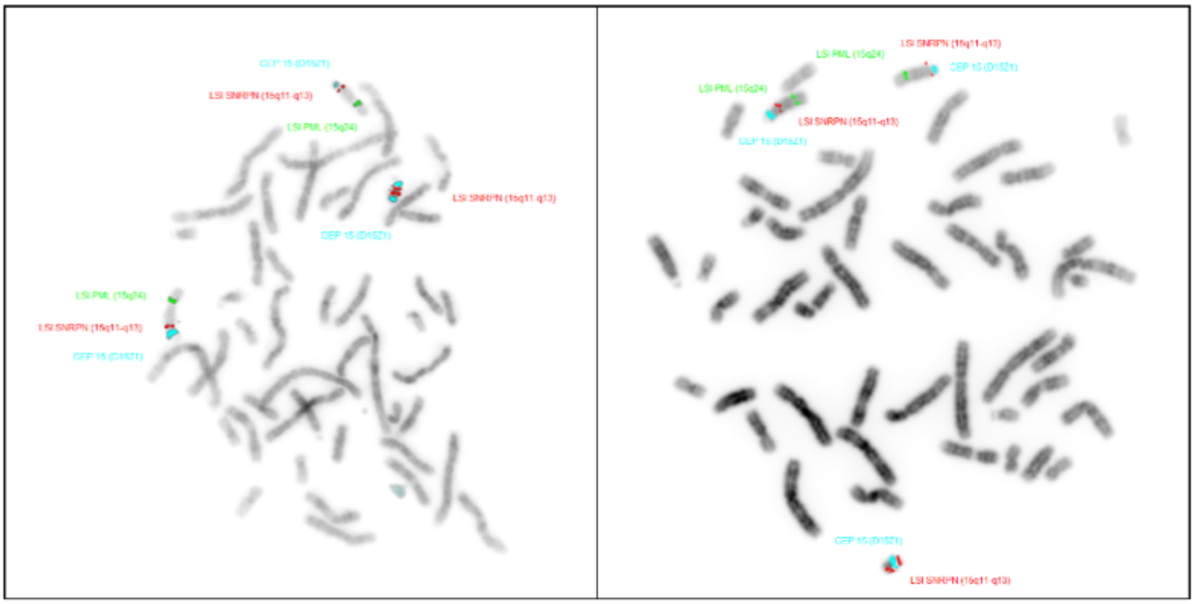

Image 1: FISH analysis of individual #3 on the left; individual #70 in the right

Two individuals were diagnosed with chromosome 15q11-q13 duplication syndrome (MIM#608636) caused by marker chromosomes. SNP-array of individual #3 revealed two additional copies of the region 15q11.1-15q13.2 (10.3 Mb) and one additional copy of the region 15q13.2-15q13.3 (1.9 Mb). In individual #70 two additional copies of the region 15q11.1-15q12 (7.8 Mb) were identified. In order to examine the conformation of the marker chromosomes, FISH analysis with Prader-Willi-/Angelman-

syndrome FISH probes was performed. Individual #3 showed the usual pattern of a dicentric chromosome. Yet, the marker chromosome of individual #70 exhibited two distal signals of the LSI-probe SNRPN and one centromere 15 signal in between, therefore an incomplete isochromosome 15. This atypical 15q Duplication Syndrome shows that only conventional chromosome analysis displays the conformation of the supernumerary chromosome and its origin.

3. *FBXO11*-associated intellectual developmental disorder with dysmorphic facies and behavioral abnormalities (MIM#618089)

Initial copy number analysis in this individual (individual #56) showed a deletion encompassing exons 5 to 10 of the *MSH6* (NM_000179.2) gene and exons 18 to 23 of the *FBXO11* (NM_025133.4) gene. Through visual inspection of the read-alignments at the region of the CNV-call using the IGV browser version 2.3.92 [8], it was possible to identify 4 read pairs with a very large insert size (Figure S2A upper panel) spanning the exons indicated as deleted by the read-depth based algorithm. The subsequent analysis of reads next to these fragments identified at least four soft-clipped reads (Figure S2A lower panel). These reads were aligned to the hg19 reference using the UCSC Genome Browser version [18] of BLAT [19] to identify the exact breakpoints. Primers spanning the deletion were designed and used for allele-specific PCR and visualized by gel electrophoresis (Figure S2B; first well DNA sample of the index, second well is an empty control for both primer pairs. Due to the large amplicon size the wildtype allele could not be amplified, leaving only the smaller variant allele.). Subsequent Sanger-sequencing of these PCR amplicons confirmed the predicted breakpoint (Figure S2C). According to BLAT alignments of the Sanger sequences (Figure S2D), we interpret the CNV to be caused by a 32 base-pair microhomology in a repeat region on both sides of the deletion (Figure S2E, F). Complete nomenclature of this 10,515 base-pair deletion, affecting both *FBOX11* and the *MSH6* genes, is: chr2:g.48030320_48040834del; NM_000179.2(MSH6):c.3173-239_*6835del, p.0?; NM_025133.4(FBXO11): c.1831+96_*4937del, p.0?.

Table S1

primer name	sequence forward	sequence reverse	wildtype	variant
AlleleSpecific_P1	GTCAGGCTGGTCCTGAACTG	CAACAAAATCTGGGGAGGA	10717bp	202bp
AlleleSpecific_P2	CAGAGTCTCTCTGTCGCCC	CCTCACGTAACATGAAGATATGG	11000bp	485bp

Figure S2

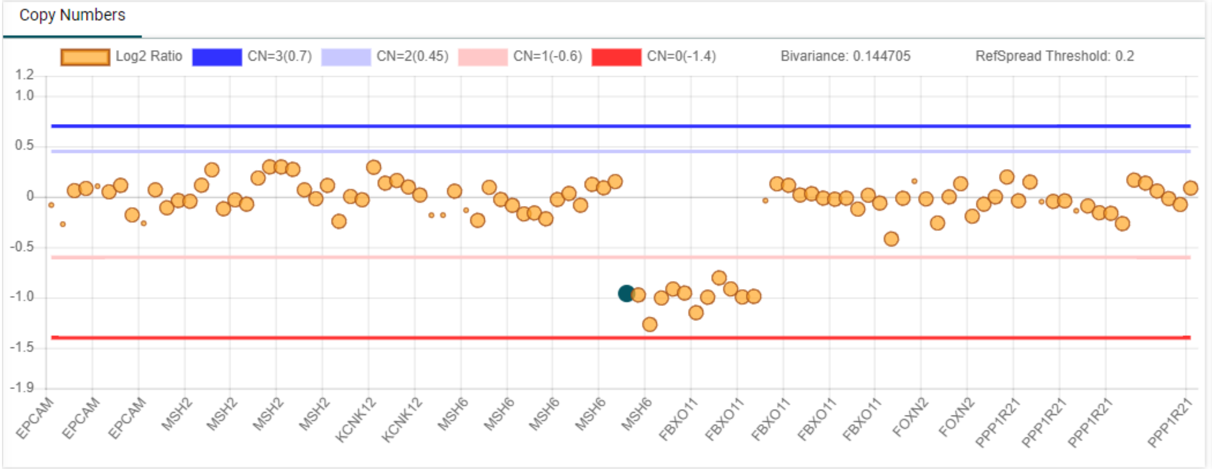

Figure S3

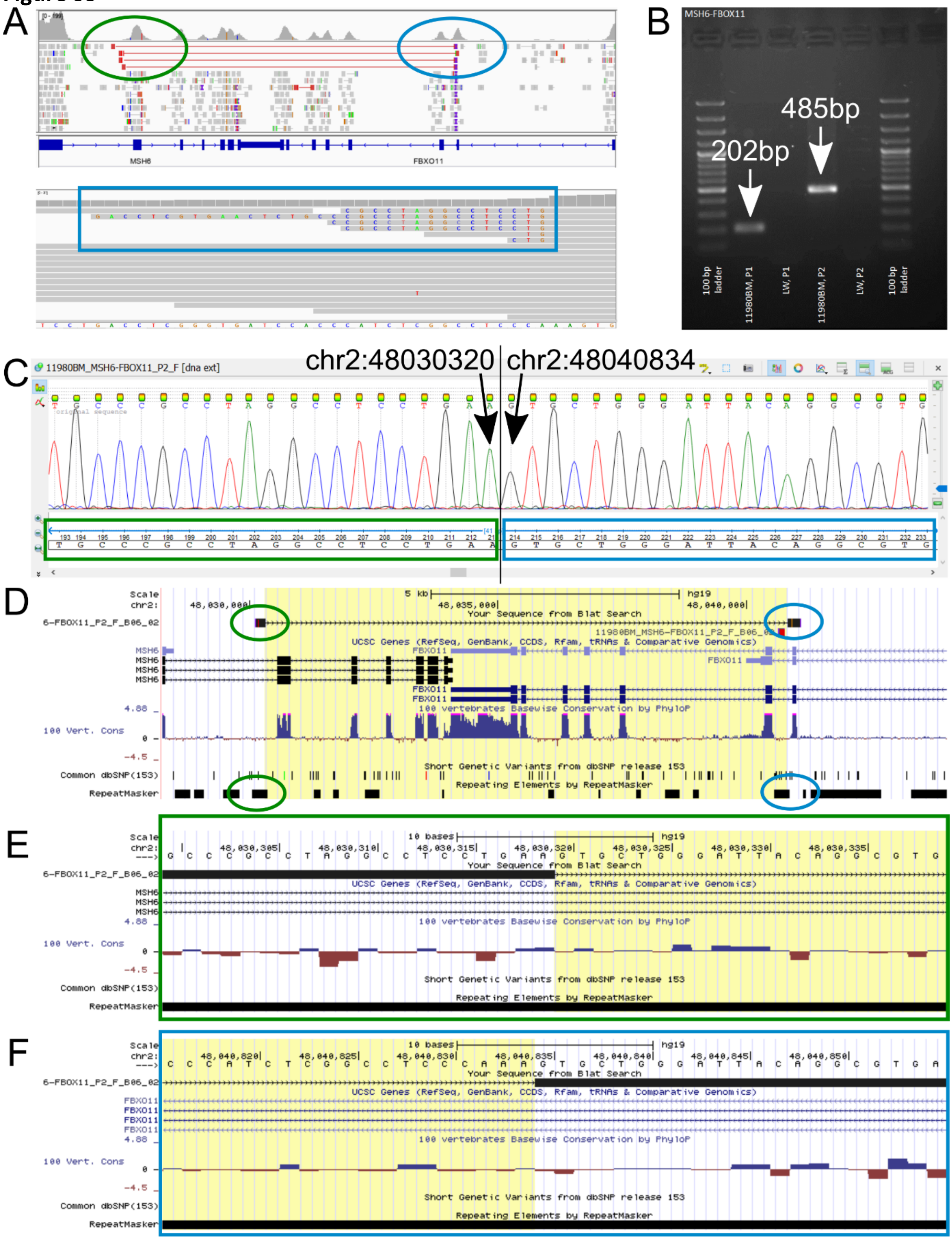
A
MSH6
FBXO11
B
MSH6-FBOX11
485bp
202bp
100 bp ladder
11980BM, P1
LW, P1
11980BM, P2
LW, P2
100 bp ladder
C
11980BM_MSH6-FBOX11_P2_F [dna ext]
chr2:48030320
chr2:48040834
D
E
F

4. Genetic diagnosis and postnatal exogenic factors

For at least two probands, a combination of a confirmed genetic diagnosis and postnatal exogenic factors may have jointly contributed to the individual phenotype. These comprise one individual with Waardenburg syndrome (MIM#193500) who was delivered preterm with severe hydrocephalus and spina bifida [20]. In 1978, at the age of 2 months, this infant received a ventriculo-peritoneal shunt. In addition to Chiari malformation and missing Falx cerebri, cerebral imaging revealed large gliotic areas at the site of the shunt, suggesting an iatrogenic lesion (individual #142).

The second individual was delivered preterm with 22q11.2 microduplication syndrome who required mechanical ventilation. Her adult phenotype includes blindness and optical atrophy, that we consider to be due to likely inappropriate peripartal oxygen administration (individual #54).

5. Cases with multiple diagnoses

Individual number/ gender/age	Disease	Combined phenotype
**#49/M, 26 years**	Microdeletion syndrome 15q13.3 (MIM#612001) caused by a heterozygous, pathogenic *de novo* 15q13.3 microdeletion (incl. one copy of *TRPM1*) of his paternal allele arr[GRCh37] 15q13.2q13.3(30371774_32514341)x1	Combined genialized and focal epilepsy with severe ID and behavioral issues, macrocephaly, obesity.
	Congenital stationary night blindness (MIM#613216) caused by a likely pathogenic, hemizygous, maternally inherited variant of *TRPM1* chr15:31340091;NM_002420.5:c.1557+1G>A; p.?	Ophthalmological phenotype with severe myopia, astigmatism and pendular nystagmus.
	Tietz albinism-deafness syndrome (MIM#103500) caused by a heterozygous, likely pathogenic, *de novo* variant in *MITF* (MIM#103500) chr3:70001032;NM_000248.3:c.629A>G;p.(Asn210Ser)	Fair skin tone, blue irises, blond hair and impaired hearing.

**#55/F, 61 years**	Epileptic encephalopathy, early infantile, 9 (MIM#300088) caused by a heterozygous likely pathogenic *PCDH19* variant chrX:99663151;NM_001184880.1:c.445C>G; p.(Pro149Ala)	Mild ID with generalized epilepsy with tonic-clonic seizures and recurrent status epilepticus in childhood as well as recurrent psychotic episodes in association with severe depressive somatization. The proband is currently free of seizures and does not require antiepileptic therapy.
	Insulin-like growth factor I, resistance to (MIM#270450) caused by a heterozygous likely pathogenic *IGF1R* variant chr15:99465432; NM_000875.4:c.2257C>T; p.(Arg753*)	Microcephaly, short stature and diabetes mellitus type II.
**#66/F, 81 years**	Epilepsy, focal, with speech disorder and with or without mental retardation (MIM#245570) caused by a likely pathogenic *GRIN2A* variant in a mosaic state (24% allele frequency in blood) chr16:9916247; NM_000833.4:c.2042G>A; p.(Arg681Gln)	Mild ID with focal epilepsy, cortical and subcortical changes of the right parahippocampal gyrus and surrounding white matter.
	Tatton-Brown-Rahman syndrome (#MIM615879) caused by a heterozygous likely pathogenic *DNMT3A* variant chr2:25471055-25471056;NM_022552.4:c.705del; p.(Glu235Aspfs*81)	Organic schizoaffective disease, obesity as well as the typical facial features with low set horizontal eyebrows and prominent upper incisors.

**#83/M, 41 years**	Dravet syndrome (MIM#607208)<br>caused by a heterozygous, pathogenic de novo *SCN1A* variant<br>chr2:166905439; NM_001165963.1:c.985G>T, p.(Gly329Cys)	Epileptologically the proband initially exhibited a classical Dravet Syndrome with recurrent convulsive status epilepticus and lack of responsiveness to antiepileptic drugs. Mild improvement on Stirpentol, Clobazam, Briveracetam and Topiramate. Significant drop of seizure frequency after adding Fenfluramine.
	Hemizygous, likely pathogenic, maternally inherited Duplication Xq28 (incl. *RAB39B, CLIC2* and *VBP1*)<br>arr[GRCh37] Xq28(154402806_154563670)x2	ID in maternal family, where a maternal uncle died at 6 years of age after being nonverbal and suffering from spastic tetraparesis, that the family referred to as "Little Syndrome".

Individuals with additional diagnosis, that they are currently asymptomatic for (detailed case description in supplement 2):

- 19-year-old female (individual #21) carried a pathogenic variant in *ARID1B* for Coffin-Siris Syndrome 1 (#135900), as well a *FMR1* premutation she is currently asymptomatic for.

Individuals with multiple diagnosis due to secondary findings (according to ACMG recommendations) [21]:

- 77-year-old male (individual #41) carried a pathogenic variant in the ACMG gene *MYPBC3*, as well a likely pathogenic variant of *SLC2A1* causative of the NDDE phenotype of Encephalopathy due to GLUT1 deficiency (#606777, #612126). The patient is currently 80 years of age and was switched to ketogenic (as by now he is receiving most food via PEG) 8 months ago. To date he shows no significant improvement of clinical symptomatology.
- 20-year-old female (individual #56) carried a deletion of exons 5-10 of the ACMG gene *MSH6*, as well as exons 18 to 23 of the neighboring Gene *FBXO11* causative of the NDDE phenotype of Intellectual developmental disorder with dysmorphic facies and behavioral abnormalities (#18089).

References

1. GenDG - Gesetz über genetische Untersuchungen bei Menschen. Accessed August 31, 2020. https://www.gesetze-im-internet.de/gendg/BJNR252900009.html#BJNR252900009BJNG000200000

2. Verma RS, Babu A. *Human Chromosomes - Manual of Basic Techniques*. Pergamon Press, New York, Oxford; 1989.

3. Firth HV, Richards SM, Bevan AP, et al. DECIPHER: Database of Chromosomal Imbalance and Phenotype in Humans Using Ensembl Resources. *The American Journal of Human Genetics*. 2009;84(4):524-533. doi:10.1016/j.ajhg.2009.03.010

4. Riggs ER, Wain KE, Riethmaier D, et al. Towards a Universal Clinical Genomics Database: The 2012 International Standards for Cytogenomic Arrays Consortium Meeting. *Human Mutation*. 2013;34(6):915-919. doi:10.1002/humu.22306

5. MacDonald JR, Ziman R, Yuen RKC, Feuk L, Scherer SW. The Database of Genomic Variants: a curated collection of structural variation in the human genome. *Nucleic Acids Res*. 2014;42(Database issue):D986-D992. doi:10.1093/nar/gkt958

6. Karczewski KJ, Francioli LC, Tiao G, et al. *Variation across 141,456 Human Exomes and Genomes Reveals the Spectrum of Loss-of-Function Intolerance across Human Protein-Coding Genes*. Genomics; 2019. doi:10.1101/531210

7. Landrum MJ, Lee JM, Benson M, et al. ClinVar: improving access to variant interpretations and supporting evidence. *Nucleic Acids Res*. 2018;46(Database issue):D1062-D1067. doi:10.1093/nar/gkx1153

8. Thorvaldsdottir H, Robinson JT, Mesirov JP. Integrative Genomics Viewer (IGV): high-performance genomics data visualization and exploration. *Briefings in Bioinformatics*. 2013;14(2):178-192. doi:10.1093/bib/bbs017

9. Schwarz JM, Rödelsperger C, Schuelke M, Seelow D. MutationTaster evaluates disease-causing potential of sequence alterations. *Nat Methods*. 2010;7(8):575-576. doi:10.1038/nmeth0810-575

10. Adzhubei I, Jordan DM, Sunyaev SR. Predicting Functional Effect of Human Missense Mutations Using PolyPhen-2. *Curr Protoc Hum Genet*. 2013;0 7:Unit7.20. doi:10.1002/0471142905.hg0720s76

11. Davydov EV, Goode DL, Sirota M, Cooper GM, Sidow A, Batzoglou S. Identifying a High Fraction of the Human Genome to be under Selective Constraint Using GERP++. Wasserman WW, ed. *PLoS Comput Biol*. 2010;6(12):e1001025. doi:10.1371/journal.pcbi.1001025

12. Pollard KS, Hubisz MJ, Rosenbloom KR, Siepel A. Detection of nonneutral substitution rates on mammalian phylogenies. *Genome Res*. 2010;20(1):110-121. doi:10.1101/gr.097857.109

13. Shapiro MB, Senapathy P. RNA splice junctions of different classes of eukaryotes: sequence statistics and functional implications in gene expression. *Nucleic Acids Res*. 1987;15(17):7155-7174. doi:10.1093/nar/15.17.7155

14. Yeo G, Burge CB. Maximum entropy modeling of short sequence motifs with applications to RNA splicing signals. *J Comput Biol*. 2004;11(2-3):377-394. doi:10.1089/1066527041410418

15. Reese MG, Eeckman FH, Kulp D, Haussler D. Improved splice site detection in Genie. *J Comput Biol*. 1997;4(3):311-323. doi:10.1089/cmb.1997.4.311

16. Desmet F-O, Hamroun D, Lalande M, Collod-Béroud G, Claustres M, Béroud C. Human Splicing Finder: an online bioinformatics tool to predict splicing signals. *Nucleic Acids Res*. 2009;37(9):e67. doi:10.1093/nar/gkp215

17. Talevich E, Shain AH, Botton T, Bastian BC. CNVkit: Genome-Wide Copy Number Detection and Visualization from Targeted DNA Sequencing. *PLoS Comput Biol*. 2016;12(4):e1004873. doi:10.1371/journal.pcbi.1004873

18. Kent WJ, Sugnet CW, Furey TS, et al. The human genome browser at UCSC. *Genome Res*. 2002;12(6):996-1006. doi:10.1101/gr.229102

19. Kent WJ. BLAT—The BLAST-Like Alignment Tool. *Genome Res*. 2002;12(4):656-664. doi:10.1101/gr.229202

20. Hart J, Miriyala K. Neural tube defects in Waardenburg syndrome: A case report and review of the literature. *Am J Med Genet*. 2017;173(9):2472-2477. doi:10.1002/ajmg.a.38325

21. on behalf of the ACMG Secondary Findings Maintenance Working Group, Kalia SS, Adelman K, et al. Recommendations for reporting of secondary findings in clinical exome and genome sequencing, 2016 update (ACMG SF v2.0): a policy statement of the American College of Medical Genetics and Genomics. *Genet Med*. 2017;19(2):249-255. doi:10.1038/gim.2016.190
